# EGFR deficiency leads to impaired self-renewal and pluripotency of mouse embryonic stem cells

**DOI:** 10.7717/peerj.6314

**Published:** 2019-01-29

**Authors:** Miaoying Yu, Yinghui Wei, Kui Xu, Shasha Liu, Lei Ma, Yangli Pei, Yanqing Hu, Zhiguo Liu, Xue Zhang, Bingyuan Wang, Yulian Mu, Kui Li

**Affiliations:** 1 State Key Laboratory of Animal Nutrition, Institute of Animal Sciences, Chinese Academy of Agricultural Sciences, Beijing, China; 2 College of Life Science, Shangrao Normal University, Shangrao, Jiangxi, China; 3 College of Life Science, Shihezi University, Shihezi, Xinjiang, China

**Keywords:** Mouse embryonic stem cells, EGFR inhibition, AG1478, Pluripotency, Self-renewal

## Abstract

**Background:**

Self-renewal and pluripotency are considered as unwavering features of embryonic stem cells (ESCs). How ESCs regulate the self-renewal and differentiation is a central question in development and regenerative medicine research. Epidermal growth factor receptor (EGFR) was identified as a critical regulator in embryonic development, but its role in the maintenance of ESCs is poorly understood.

**Methods:**

Here, EGFR was disrupted by its specific inhibitor AG1478 in mouse ESCs (mESCs), and its self-renewal and pluripotency were characterized according to their proliferation, expression of pluripotency markers, embryoid body (EB) formation, and mRNA expression patterns. We also used another EGFR inhibitor (gefitinib) and RNA interference assay to rule out the possibility of non-specific effects of AG1478.

**Results:**

EGFR inhibition by AG1478 treatment in mESCs markedly reduced cell proliferation, caused cell cycle arrest at G_0_/G_1_ phase, and altered protein expressions of the cell cycle regulatory genes (CDK2 (decreased 11.3%) and proliferating cell nuclear antigen (decreased 25.2%)). The immunoreactivities and protein expression of pluripotency factors (OCT4 (decreased 26.9%)) also dramatically decreased, while the differentiation related genes (GATA4 (increased 1.6-fold)) were up-regulated in mESCs after EGFR inhibition. Meanwhile, EGFR inhibition in mESCs disrupted EB formation, indicating its impaired pluripotency. Additionally, the effects observed by EGFR inhibition with another inhibitor gefitinib and siRNA were consistent with those observed by AG1478 treatment in mESCs. These effects were manifested in the decreased expression of proliferative and pluripotency-related genes and the increased expression of genes involved in differentiation. Moreover, RNA-seq analysis displayed that transcript profiling was changed significantly after EGFR inhibition by AG1478. A large number of differentially expressed genes were involved in cell cycle, apoptotic process, epigenetic modification, and metabolic process, which were related to self-renewal and pluripotency, confirming that EGFR deficiency impaired self-renewal and pluripotency in mESCs.

**Conclusions:**

Taken together, our results demonstrated the importance of EGFR in guarding the stemness of mESCs.

## Introduction

Embryonic stem cells (ESCs) are derived from the inner cell mass of preimplantation blastocysts, with unlimited self-renewal capacity. These pluripotent cells can differentiate into various cell types representing all the three germ layers to participate in development and formation of an adult body (Evans & Kaufman, 1981; Martin, 1981). A better understanding of the molecular mechanisms regulating ESCs property is inevitably and urgently required to exploit their utilization toward full potential and safety. In vitro cultured ESCs are ready to switch from self-renewal state to multi-differentiation during their proliferation. Fate determination of ESCs is regulated by the cross-talk between extraneous stimulus signals and interior gene expression profile (Pollard et al., 2008; Martello et al., 2012; Saiz & Plusa, 2013; Hall et al., 2009; Schrode et al., 2013). Self-renewal is intrinsically associated with cell cycle control. The cell cycle in pluripotent ESCs is governed by unique mechanisms that support unrestricted proliferation and competency for appropriate differentiation. As the choice between self-renewal and differentiation, the pluripotency state of ESCs is stabilized by an integration of stimulus upon interconnected network of pluripotency factors (Chen et al., 2008; Pollard et al., 2008; Hall et al., 2009; Ng & Surani, 2011; Wray et al., 2011). Pluripotency factors, especially OCT4, Nanog, and SOX2, play central roles in controlling self-renewal and preventing onset of differentiation. The down-regulation of these three core pluripotency factors induces ESCs differentiation (Filipczyk et al., 2013; Rizzino, 2013; Young, 2011). The self-renewal activity of ESCs is directly connected with their unique short G_1_ phase, and most cells are in S phase at any given time (Stead et al., 2002; White & Dalton, 2005). Expression changes of cell-cycle regulatory proteins and cell-cycle structure were demonstrated to be associated with differentiation initiation. Factors controlling the cell cycle progress might be necessary to maintain pluripotency upon block differentiation (Viatour, 2012). Common regulation genes and pathways were suggested in both cell cycle and self-renewal activities by recent literature (White et al., 2005; Zhang et al., 2009). Many cell cycle related factors were detected under regulation of pluripotency factors such as OCT4, SOX2, Nanog, and Lin28, including CDK1, cyclin D1, CDK6, CDC25A, CDC7, cyclinA, cyclinB, and CDK4 (Boyer et al., 2005; Yu et al., 2007; Hanna et al., 2009; Prenzel et al., 2001).

It is well-documented that mouse ESCs (mESCs) retain self-renewal and pluripotency in the presence of leukaemia inhibitory factor (LIF) and bone morphogenetic protein-4 (Hall et al., 2009; Hirai, Karian & Kikyo, 2011; Ying et al., 2003). LIF-LIFR/gp130-JAK-STAT3 pathway provides activation of pluripotency factors expression and down-regulation of differentiation related genes (Ying et al., 2003; Cartwright et al., 2005; Niwa et al., 2009; Hall et al., 2009). However, the precise mechanisms coordinating cell fate determination and pluripotency remain poorly understood. It is not fully uncovered how the balance between self-renewal and differentiation is regulated.

Epidermal growth factor receptor (EGFR) plays critical roles in many cellular progresses, including proliferation, differentiation and apoptosis (Prenzel et al., 2001). EGFR was reported to be the upstream regulator critical for STAT3 phosphorylation in cancer cells and cancer stem- like cells (Cheng et al., 2018; Park et al., 2018; Ta et al., 2018). EGFR and its ligand EGF were reported to be highly expressed in embryos, and EGFR knockout in mice led to preimplantation death of the embryos, suggesting that the EGF-EGFR system might be related to early embryo development in an autocrine and/or paracrine manner (Kim et al., 1999; Lee et al., 2005; Paria et al., 1994; Dadi, Li & Lloyd, 2009). RNA interference assay demonstrated that down-regulation of EGFR and EGF expression impaired survival of embryos and delayed implantation (Ellis et al., 2006). The role of EGFR in mESCs still remains poorly understood. In this study, chemical inhibitors of EGFR and RNA interference were adopted to investigate the effect of EGFR deficiency on mESCs self-renewal and pluripotency, and a RNA-seq was used to demonstrate the global gene expression change in EGFR deficient mESCs.

## Materials and Methods

### Cell culture and experimental design

E14.1 mESC (ATCC, Manassas, VA, USA) were cultured in Dulbecco’s modified Eagle’s medium (Gibco, Grand Island, NY, USA), supplemented with one mM sodium bicarbonate (Gibco), 1% penicillin/streptomycin (Gibco), two mM L-glutamine (Gibco), 1% MEM non-essential amino acids (Gibco), 0.1 mM β-mercaptoethanol (Sigma, Darmstadt, Germany), 1,000 U/ml mouse LIF (Millipore, Darmstadt, Germany), one μM PD0325901 (Selleck, Houston, TX, USA), three μM CHR99021 (Selleck), and 15% (v/v) ES cell-qualified fetal bovine serum (Millipore). Cells were cultured on 0.1% gelatin coated 6-well plate at a density of 10^6^ cells per well, at 37 °C and an atmosphere of 5% CO_2_. For embryoid body (EB) formation, cells were cultured in drop-culture method with a 25 μl drop containing 1,000 cells, without LIF and 2i (PD0325901 and CHR99021). Cells were divided into two groups, EGFR inhibition group and control group. For EGFR inhibition group, mESCs were treated with 5, 10, 15, and 20 μΜ AG1478 (Selleck) for 24 h, and the concentration of 10 μΜ was the most suitable (Figs. S2A–S2E). Additionally, mESCs were treated with 0.05, 0.5, 5, and 10 μΜ gefitinib (Selleck) for 24 h, and the concentration of five μΜ was the most suitable (Fig. S3A). The stock solution for inhibitors used were prepared in dimethyl sulfoxide.

### Cell proliferation assay and cell cycle analysis

Mouse ESCs were seeded in 0.1% gelatin coated 96-well plates at a cell density of 5 × 10^3^ per well with treatment of AG1478 or not. Cell Counting kit-8 Kit (CCK-8) (Dojindo Molecular Technologies, Rockville, MD, USA) was used to compare cell proliferation according to the manufacturer’s instructions. Cells were fixed in 70% ice-cold ethanol at 4 °C overnight, then incubated with 0.1 mg/ml RNase A (Sigma) for 30 min followed by incubation with 100 μg/ml propidium iodide (PI) (Sigma) staining for 5 min at room temperature and avoid light condition. Cell-cycle analysis was performed immediately using flow cytometry.

### Alkaline phosphatase staining

Expression of alkaline phosphatase (AP) was detected by staining with an AP detection kit (Stemgent, Cambridge, MA, USA) following manufacturer’s instructions.

### Immunofluorescence

Cells were fixed with 4% paraformaldehyde in PBS for 20 min at room temperature. To detect intracellular antigens, cells should be permeabilized with 0.1% Triton X-100 after fixation. Cells were then blocked by 5% BSA in PBS at room temperature for 30 min, and were incubated with primary antibodies for EGFR (1:500; CST, Danvers, MA, USA), SSEA-1 (1:500; Santa Cruz Biotechnology, Dallas, TX, USA), OCT4 (1:500; CST), and Nanog (1:500; CST) overnight at 4 °C. Cells were incubated with fluorescence-conjugated secondary antibodies for 1 h at room temperature and avoid from light. Nuclei were stained with 4′,6-diamidino-2-phenylindole, Fluorescence signals were detected with a fluorescence microscope.

### Western blotting

Cells were lysed using T-PER^®^ Protein Extraction Reagent (Thermo-Fisher, Waltham, MA, USA) containing protease inhibitor cocktail (Roche, Basel, Switzerland). Equal amount (30 μg per lane) of denatured proteins were separated by SDS–PAGE and transferred to nitrocellulose membranes (Millipore). Then membranes were blocked with 5% non-fat milk for 2 h at room temperature, and then incubated with the primary antibodies (CST) of recommended concentration diluted in 5% non-fat milk overnight at 4 °C. HRP-conjugated anti-rabbit IgG was used as secondary antibody and the blots were developed using the Pierce ECL Western Blotting Substrate according to the manufacturer’s instructions (Pierce, Appleton, WI, USA). The visualized bands were quantified by calculating net light density value with Gel Image System 1D software (v4.2), based on a Tanon-5200 Chemiluminescent Imaging System (Shanghai, China).

### Targeting knock-down assays

For transient short interfering RNA (siRNA) transfection, cells at 70% confluence were transfected using Lipofectamine^®^ RNAiMAX (Thermo-Fisher, Waltham, MA, USA) in complete medium according to the manufacturer’s instructions, with a final siRNA concentration of 100 nM. The siRNAs were incubated with Lipofectamine^®^ RNAiMAX reagent for approximately 20 min and diluted with Opti-MEM^®^ medium. The siRNA-lipid complex was gently added to the culture medium. A total of 6 h later, the culture medium was replaced with fresh medium. A total of 48 h after transfection cells were trypsinized and plated for the different experiments. siRNAs were purchased from GenePharma (http://www.genepharma.com/). siRNA sequences were as follows: EGFR siRNA-1: 5′-CUGUGCGAUUCAGCAACAA-3′; EGFR siRNA-2: 5′-CCACCUAUCAGAUGGAUGU-3′; EGFR siRNA-3: 5′-CCCUGUCGCAAAGUUUGUA-3′; EGFR siRNA-4: 5′-GUGCUACGCAAACACAAUA-3′; and control non-targeting siRNA-NC: 5′-UUCUCCGAACGUGUCACGU-3′.

### RNA preparation, libraries sequencing, and reads mapping

Total RNAs were isolated from mESCs in EGFR inhibition group and control group, using Trizol reagent (Invitrogen, Carlsbad, CA, USA) according to the manufacturer’s instructions. RNA degradation and contamination was monitored by 1% agarose gels. RNA purity was checked by NanoDrop ND-1000 spectrophotometer (IMPLEN, Westlake Village, CA, USA). RNA integrity was assessed using RNA Nano 6000 Assay Kit of the Bioanalyzer 2100 system (Agilent Technologies, Santa Clara, CA, USA). A total amount of three μg RNA of each mESCs sample (from EGFR inhibition group or control group) was used to construct cDNA library for RNA-seq analysis, after ribosomal RNA removal. Subsequently, sequencing libraries were generated using the rRNA-depleted RNA by NEBNext^®^ Ultra™ Directional RNA Library Prep Kit for Illumina^®^ (NEB, Ipswich, MA, USA). The constructed libraries were sequenced on an Illumina HiSeq2500 platform (FC-104-5001; Illumina, San Diego, CA, USA). The RNA-seq experiments were performed by Experimental Department of Novogene Corporation (Beijing, China).

Clean reads were obtained from raw reads after the removal of reads containing an adapter, reads with low quality, and reads containing ploy-N. At the same time, Q20, Q30, and GC content were calculated for the clean dataset. All the down stream analyses were based on the clean data. Clean reads were aligned to the mouse genome mm10 by Tophat 2.0.3 (Kim et al., 2013) with default parameters. Only the uniquely mapped reads were used for future expression analysis. HTSeq quantified RNAs based on annotation with the union model, and distributed reads into different known types of genes.

### Gene expression and functional annotation

The mapped reads of each sample were assembled by both Scripture (beta2) (Guttman et al., 2010) and Cufflinks software (v2.1.1) (Trapnell et al., 2010) in a reference-based approach. Cufflinks was used to calculate fragments per-kilo-base of exon per million fragments mapped (FPKM) of genes in each sample to quantify their expression levels. Based on FPKM of each replicate, Pearson’s correlation coefficient (*R*^2^) was calculated to measure the correlation between two variables. Cuffdiff (http://cole-trapnell-lab.github.io/cufflinks/) was used to identify significant differentially expressed genes (DEGs) between the two mESCs groups, and corrected *P*-value <0.05 was set as the threshold for DEGs. Among these DEGs, nine protein coding genes were randomly selected for quantitative PCR to validate the mRNAs expression results from RNA-seq, and data are presented as the log_2_ fold change between EGFR inhibition and control mESCs.

Differentially expressed genes were classified according to gene ontology (GO) enrichment analysis and kyoto encyclopedia of genes and genomes (KEGG) analysis. The GO categories were derived from (http://www.geneontology.org), and GO enrichment of DEGs was implemented by the GO seq R package (Langfelder & Horvath, 2008; Pennisi, 2012; Young et al., 2010) in which gene length bias was corrected. GO terms with corrected *P*-value < 0.05 were considered significantly enriched by DEGs, based on Wallenius non-central hyper geometric distribution. KEGG is a database resource for understanding high-level functions and utilities of the biological system (http://www.genome.jp/kegg/) (Kanehisa et al., 2008). Each KEGG pathway will be considered as a unit gene set. And geometric test will interrogate significantly enriched pathway based on background genes and DEGs list. We used KOBAS (2.0) software (Mao et al., 2005) to test the statistical enrichment of differential expression genes in KEGG pathways, and corrected *P*-value <0.05 were considered significantly enriched by DEGs.

### Quantitative PCR

Total RNAs were reverse transcribed using First Strand cDNA Synthesis Kit (Thermo Scientific, Waltham, MA, USA) according to the manufacturer’s instructions, and were used for quantitative PCR with SYBR Green master mix on an ABI PRISM 7500 Fast Real-Time PCR system (Applied Biosystems, Foster City, CA, USA) as previously described (Tao et al., 2015). Gene specific primers were designed using Primer3 software32 (Tables S1–S4). In addition, reverse transcription-polymerase chain reaction (RT-PCR) primer for EGFR was as follows: forward: 5′-ATCACAATCAGCCCCTGCAT-3′ and reverse 5′-TGCCATTTGGCTTGGTTTCC-3′. Quantitative data were normalized to GAPDH, and the relative quantity was calculated using the (2^−ddCt^) method.

### Statistical analysis

Experiments were performed independently at least three times. Numerical data were expressed as the means ± SD. Statistical analysis was performed using SPSS 19 software. Differences between two groups were assessed using a Student’s *t*-test. *P*-value < 0.05 was considered to be statistically significant difference.

## Results

### EGFR inhibition hinders proliferation of mESCs by inducing cell cycle arrest at G_0_/G_1_ phase

**Figure 1:**
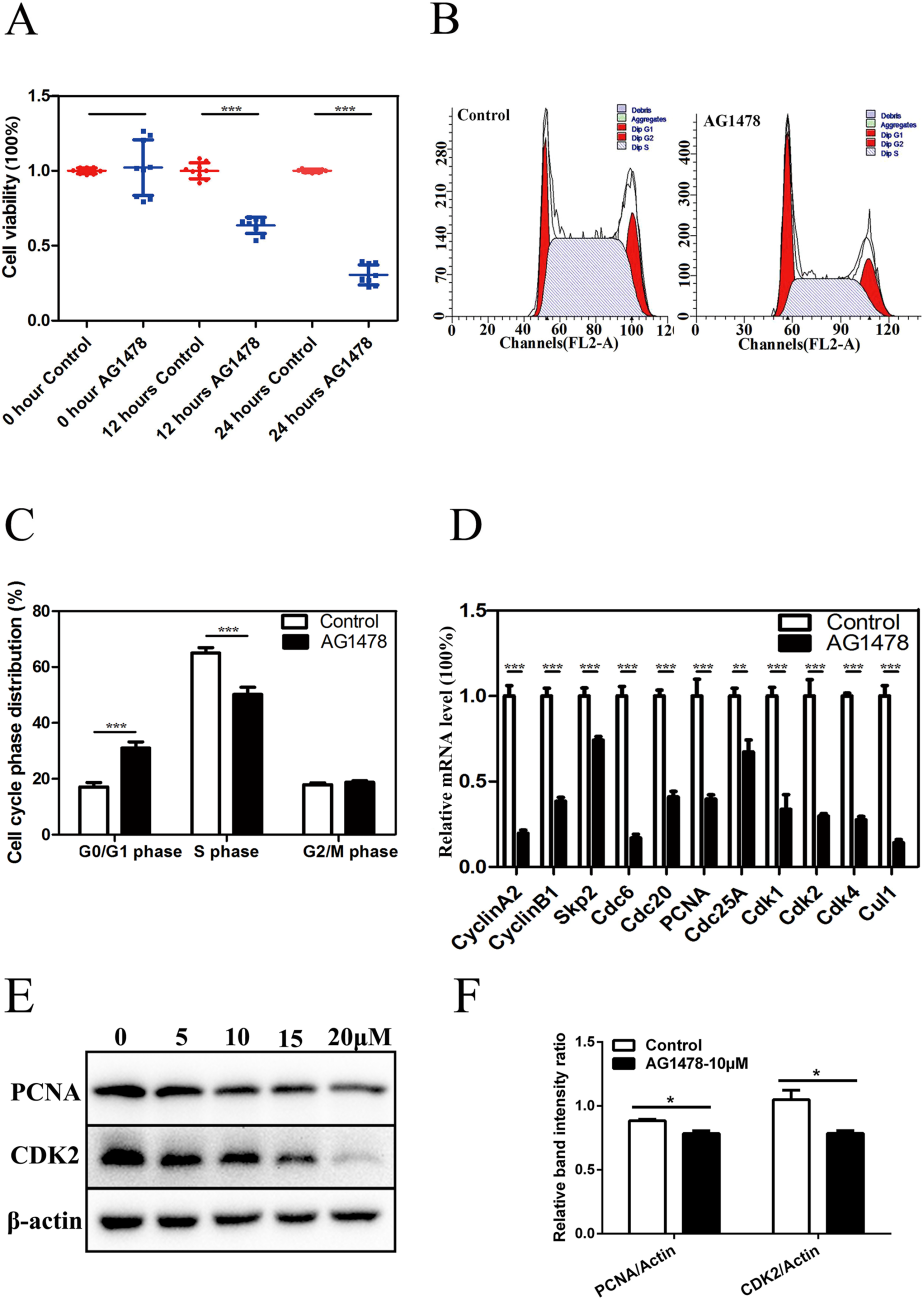
Inhibition of EGFR by AG1478 hinders mESCs proliferation and induces cell cycle arrest at G_0_/G_1_ phase. (A) Control and AG1478 treated mESCs were compared for cell viability by CCK-8. (B and C) Cell cycle phase distribution of control and AG1478 treated mESCs. (D) Quantitative PCR analysis of mRNA levels of cell cycle regulatory genes in control and AG1478 treated mESCs. The amounts of each mRNA were normalized to GAPDH mRNA and are shown relative to the amounts of control mESCs (set to 1). (E) Protein expression of cell cycle regulatory genes (CDK2 and PCNA) in control and AG1478 treated mESCs. β-actin served as a loading control. (F) Quantified relative band intensity ratio of CDK2 and PCNA. The data are presented as mean ± SD (*n* = 3; *P < 0.05, **P < 0.01, ***P < 0.001, Student’s *t*-test).

Immunofluorescent staining and RT-PCR results showed that EGFR was expressed in mESCs (Figs. S1A and S1B). CCK-8 assay showed that the cell viability was significantly lower in AG1478 treated mESCs than control cells, which suggests that EGFR deficiency hinders mESCs proliferation (Fig. 1A). To address how EGFR affects mESCs proliferation, we analyzed cell cycle distribution of AG1478 treated mESCs by Flow cytometry analysis. Relative to control mESCs, the proportion of G_0_/G_1_ phase in AG1478 treated mESCs was increased significantly by approximately 81.72%, while the proportion of S phases was decreased significantly by approximately 22.76% (Figs. 1B and 1C). These results suggested that EGFR inhibition could hinder mESCs proliferation by arresting cell cycle at G_0_/G_1_ phase. Quantitative PCR and western-blot were used to examine the expressions of pivotal cell cycle regulatory genes. EGFR-deficient mESCs showed decreased mRNA expressions of CDK2 (decreased 70.2%), CDK4 (decreased 72.5%), and proliferating cell nuclear antigen (PCNA) (decreased 60.3%) (Fig. 1D). Meanwhile, EGFR-deficient mESCs showed decreased protein expressions of CDK2 (decreased 11.3%) and PCNA (decreased 25.2%) (Figs. 1E and 1F). Thus, EGFR inhibition by AG1478 arrested mESCs cell cycle at G_0_/G_1_ phase through altering the expression of pivotal cell cycle regulatory genes.

### EGFR deficiency impairs mESC self-renewal and pluripotency and drives mESC differentiation

**Figure 2:**
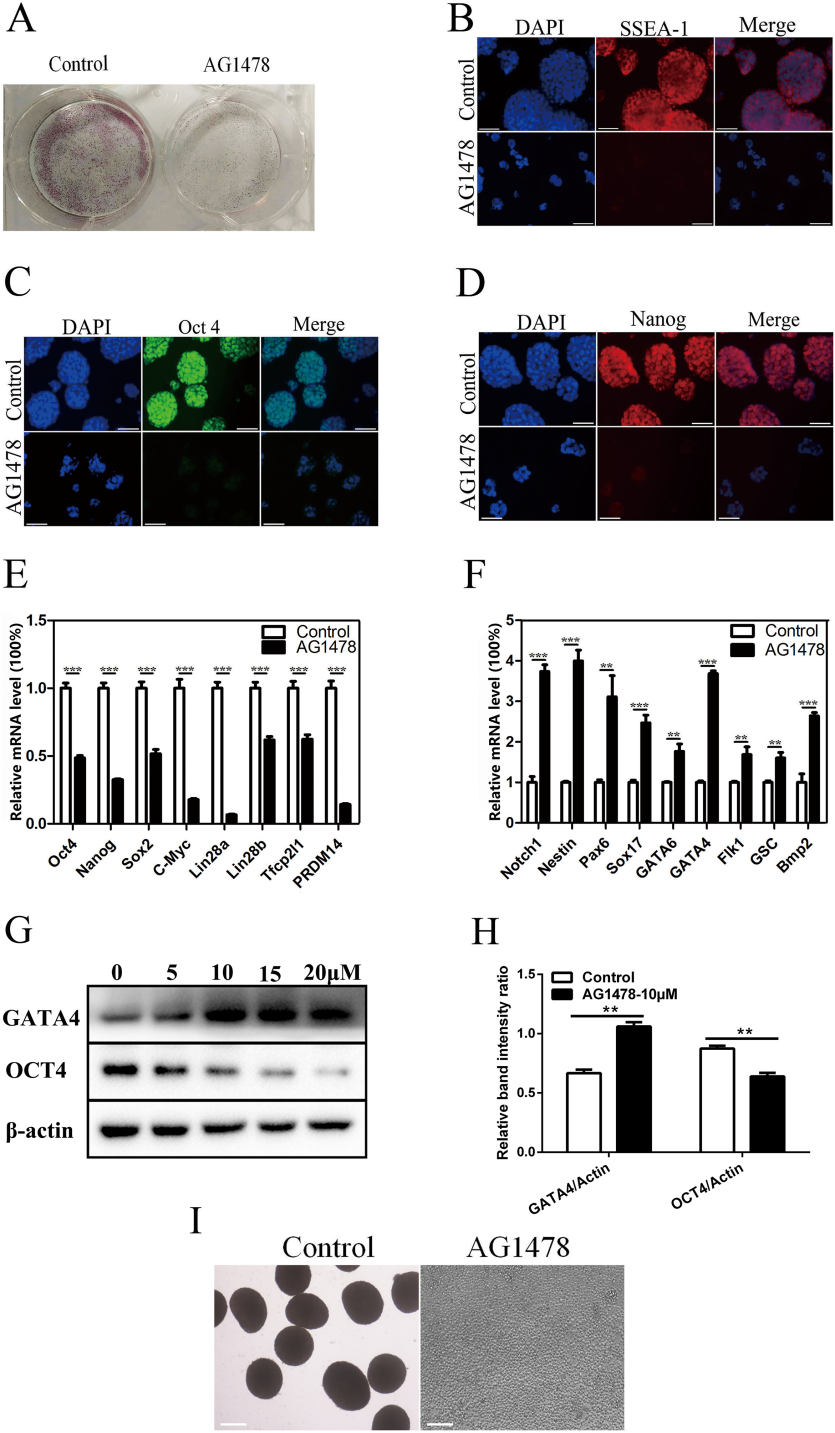
Inhibition of EGFR by AG1478 impairs mESC self-renewal and pluripotency, and induces differentiation. (A) Enzymatic activity for AP was analyzed in control and AG1478 treated mESCs. (B–D) IF staining against SSEA-1 (red), OCT-4 (green), and Nanog (red) in control and AG1478 treated mESCs. Nuclei were counterstained by 4′,6-diamidino-2-phenylindole, scale bar: 200 μm. (E and F) Quantitative PCR analysis of mRNA levels of pluripotency factor s and differentiation related genes in control and AG1478 treated mESCs. The amounts of each mRNA were normalized to GAPDH mRNA and are shown relative to the amounts in control mESCs (set to 1). (G) Protein expression of OCT4 and GATA4 in control and AG1478 treated mESCs. β-actin served as a loading control. (H) Quantified relative band intensity ratio of OCT4 and GATA4. The data are presented as mean ± SD (*n* = 3; **P < 0.01, ***P < 0.001, Student’s *t*-test). (I) The performance of EB formation of control and AG1478 treated mESCs, scale bar: 100 μm.

Epidermal growth factor receptor inhibition resulted in a dramatic reduction in the number and size of AP positive mESC colonies (Fig. 2A), and a decreased expression of stage-specific embryonic antigen 1 (SSEA-1) on mESC surface (Fig. 2B). The reduction in the immunoreactivity of two pluripotency markers implied the reduced pluripotency state and impaired self-renewal in EGFR-inhibited cells. EGFR inhibition also reduced the expression of pluripotency factors like OCT4 (decreased 51.5% in mRNA level, and decreased 26.9% in protein level) and Nanog (decreased 67.5%) (Figs. 2C–2E, 2G and 2H), and impaired pluripotency protection network composed of several key factors including Lin28b (decreased 38.2%) and PRDM14 (decreased 85.7%) (Fig. 2E), suggesting that EGFR is necessary for maintaining mESC pluripotency. To investigate whether EGFR inhibition would induce mESC differentiation, we examined the expression levels of early differentiation markers by quantitative PCR and western-blot (Figs. 2F–2H). As a result, EGFR inhibition led to an increase in the mRNA levels of three germ layer markers including Notch1 (increased 3.7-fold), Nestin (increased 4.0-fold), and Pax6 (increased 3.1-fold) (Fig. 2F), suggesting that EGFR inhibition drives mESC differentiation. EB formation reflects the pluripotency of mESCs. Here, we found that EB formation failed in mESCs after EGFR inhibition by AG1478, confirming the impairment of pluripotency in EGFR inhibited mESCs (Fig. 2I).

### Gefitinib and RNA interference induced similar gene expression changes as AG1478 treatment in mESCs

**Figure 3:**
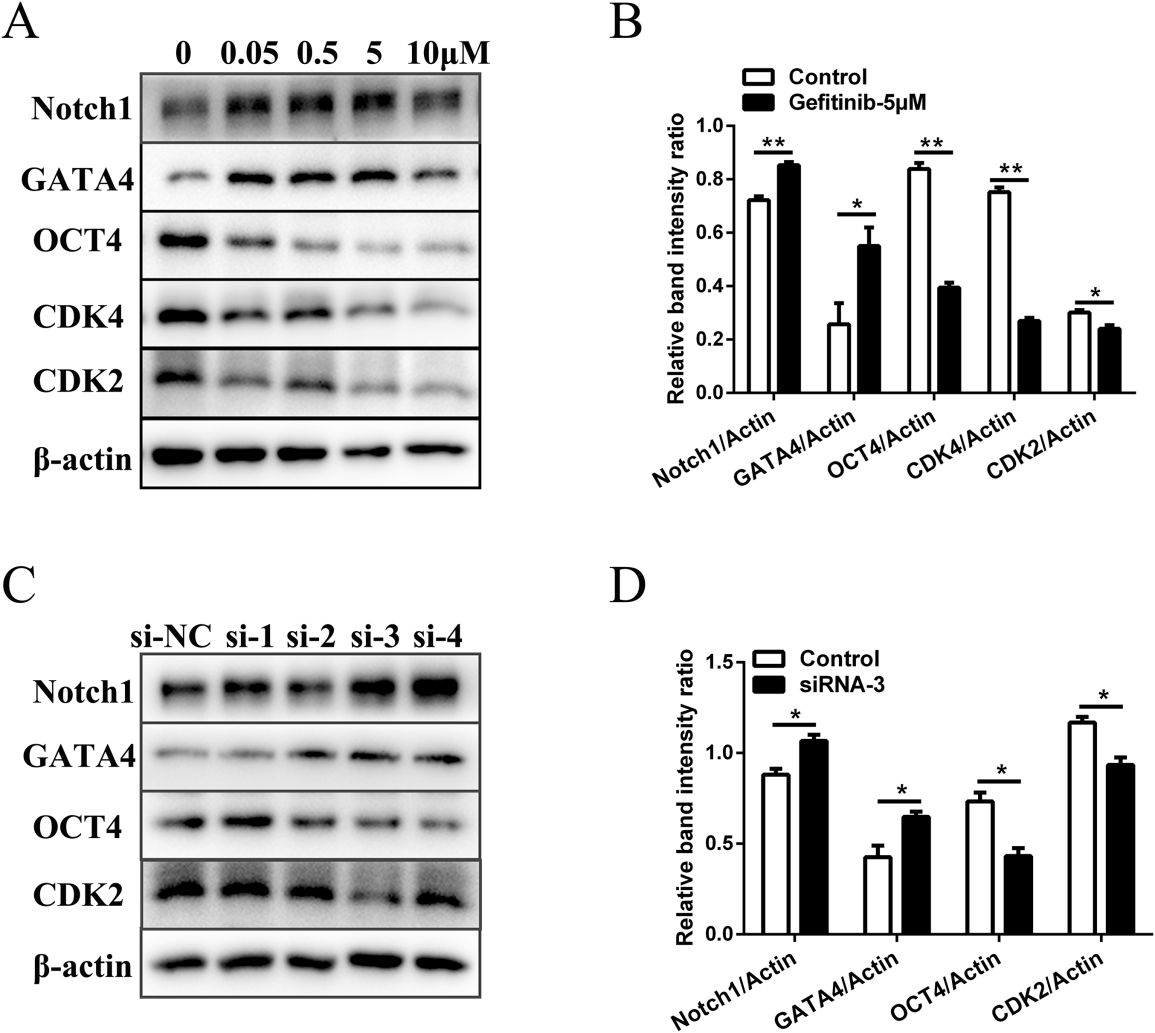
Inhibition of EGFR by gefitinib and RNA interference impairs mESCs cell cycle and self-renewal related genes expression. (A) Protein expression of cell cycle regulatory genes (CDK2 and CDK4), pluripotency factor (OCT4), and differentiation related genes (GATA4 and Notch1) in control and gefitinib treated mESCs. β-actin served as a loading control. (B) Quantified relative band intensity ratio of A. (C) Protein expression of cell cycle regulatory genes (CDK2), pluripotency factor (OCT4), and differentiation related genes (GATA4 and Notch1) in mESCs after RNA interference with different siRNAs. β-actin served as a loading control. (D) Quantified relative band intensity ratio of C. The data are presented as mean ± SD (*n* = 3; *P < 0.05, **P < 0.01, Student’s *t*-test).

To rule out the possibility of non-specific effects of AG1478, we also used another EGFR inhibitor (gefitinib) and RNA interference assay to analyze the proliferation, differentiation, and expression of pluripotency markers of mESCs. Five μM was selected as the suitable concentration to inhibit EGFR phosphorylation activity (Fig. S3A). EGFR inhibition by gefitinib in mESCs markedly reduced protein expressions of the cell cycle regulatory genes (CDK2 (decreased 20.1%) and CDK4 (decreased 64.1%)). The protein expression of pluripotency factors (OCT4 (decreased 52.9%)) also dramatically decreased, while the differentiation related genes (GATA4 (increased 2.1-fold) and Notch1 (increased 1.2-fold)) were up-regulated in mESCs after EGFR inhibition (Figs. 3A and 3B). Four siRNA oligonucleotides were prepared and EGFR expression was knocked down after RNA interference (Fig. S3B). Western-blot detection revealed that the protein expressions of CDK2 (decreased 20.0%) and OCT4 (decreased 41.2%) were reduced, while the protein expressions of GATA4 (increased 1.5-fold) and Notch1 (increased 1.2-fold) were elevated in mESCs by silencing of the EGFR with siRNA-3 (Figs. 3C and 3D). These genes were related to cell cycle, self-renewal, pluripotency, and differentiation. The effects observed by EGFR inhibition with another inhibitor gefitinib and siRNA were consistent with those observed by AG1478 treatment in mESCs. Thus, we further confirmed the on-target effects of AG1478 on mESCs including cell cycle progress, self-renewal and pluripotency through EGFR impairment.

### EGFR inhibition causes transcriptional changes involved in self-renewal and pluripotency

We sequenced cDNA libraries from three AG1478 treated mESCs samples (numbered as Treat 1, Treat 2, and Treat 3) and three control mESCs samples (numbered as Control 1, Control 2, and Control 3). In total, 746,337,642 raw reads were acquired with an error rate 0.02%, and then 734,603,314 clean reads were generated from raw reads (Table S5). Approximately 611,878,157 clean reads were mapped to the mouse genome mm10, and the alignment percentage for each sample was more than 81.24%. The percentage of uniquely mapped reads was more than 69.32% for each sample (Table S6). The Pearson correlation coefficients of gene expression levels were greater than 0.95 in both EGFR inhibited group and the control group (Fig. S1C), demonstrating the similarity of expression within one group, the rationality of samples selection, and the reliability of sequencing data.

**Figure 4:**
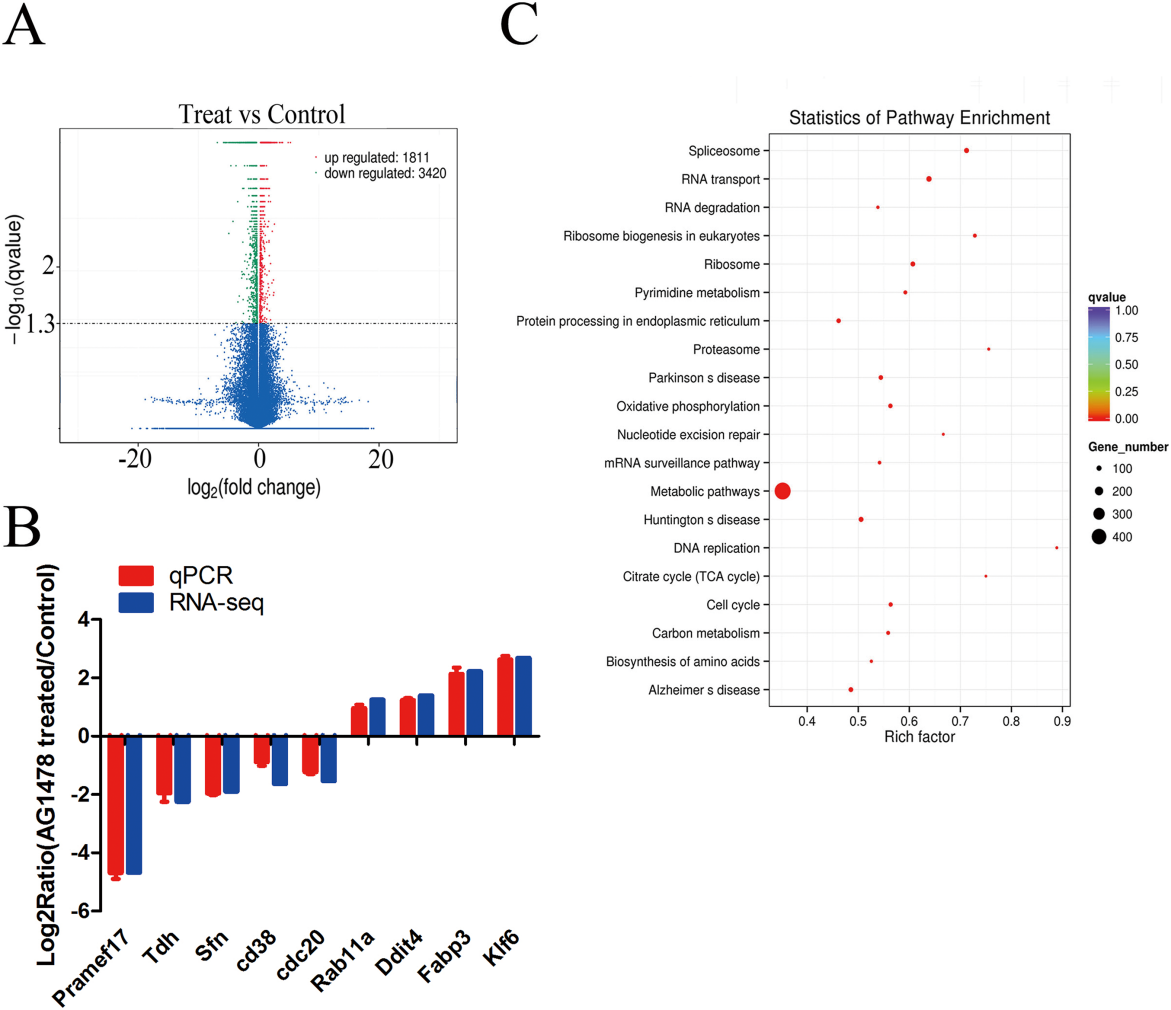
Differentially expressed genes and KEGG pathway enrichment. (A) Volcano plot of the identified genes in all biological replicates (the genes that showed significant down- and up-regulated after statistical analysis are reported in green and red). (B) Validation of RNA-Seq data by quantitative PCR. Each gene was normalized to GAPDH. Data are presented as the log_2_ fold change between EGFR inhibition and control mESCs. (C) Scatter plot of top 20 KEGG enrichments of mRNA. Vertical coordinate represents pathway name, and horizontal coordinate represents Rich factor. The size and color of points represent the number of differentially expressed genes in the pathway and the range of different corrected *P*-values, respectively.

The expression profiling showed global significant changes in gene expression between two mESCs groups. 5,231 genes were considered as significant differentially expressed with corrected *P*-value <0.05, including mRNAs and lncRNAs. Among them, 1,811 genes were up-regulated and 3,420 genes were down-regulated in response to EGFR inhibition (Fig. 4A). And the transcription changes of mRNAs were analyzed in the present article. Quantitative PCR validation results of nine randomly selected genes were consistent with RNA-seq data, which are related to cell cycle (Sfn, Cdc20, Rab11a), p53 signaling pathway (Sfn), ribosome (Pramef17, Klf6), citrate cycle (TCA cycle) (Tdh), and oxidative phosphorylation (Ddit4) (Fig. 4B). Through GO survey, we observed several self-renewal and pluripotency associated terms, such as cell cycle, apoptotic process, stem cell maintenance, condensed chromosome, chromatin binding, histone binding, and transcription factor binding (Table 1). As determined by KEGG analysis, many DEGs were enriched in pathways related to self-renewal and pluripotency such as cell cycle, p53 signaling pathway, ribosome, Citrate cycle (TCA cycle), and oxidative phosphorylatio (Fig. 4C; Table 2).

**Table 1:** Self-renewal and pluripotency related GO categories significantly enriched from DEGs between two mESCs groups.

GO ID	GO category	Description	Corrected *P*-value
GO:0007049	Biological process	Cell cycle	3.14*E*-53
GO:0000075	Biological process	Cell cycle checkpoint	3.75*E*-13
GO:0022403	Biological process	Cell cycle phase	1.75*E*-05
GO:0044770	Biological process	Cell cycle phase transition	3.22*E*-19
GO:0022402	Biological process	Cell cycle process	2.51*E*-48
GO:0000278	Biological process	Mitotic cell cycle	3.99*E*-38
GO:0007093	Biological process	Mitotic cell cycle checkpoint	2.01*E*-08
GO:0044772	Biological process	Mitotic cell cycle phase transition	3.55*E*-16
GO:0045786	Biological process	Negative regulation of cell cycle	0.001747
GO:1901988	Biological process	Negative regulation of cell cycle phase transition	1.72*E*-12
GO:0010948	Biological process	Negative regulation of cell cycle process	2.93*E*-11
GO:0051726	Biological process	Regulation of cell cycle	7.67*E*-20
GO:1901987	Biological process	Regulation of cell cycle phase transition	1.06*E*-16
GO:0007346	Biological process	Regulation of mitotic cell cycle	2.24*E*-09
GO:1901990	Biological process	Regulation of mitotic cell cycle phase transition	9.37*E*-13
GO:0000082	Biological process	G1/S transition of mitotic cell cycle	0.000163
GO:1901989	Biological process	Positive regulation of cell cycle phase transition	0.003147
GO:0090068	Biological process	Positive regulation of cell cycle process	0.000392
GO:0006915	Biological process	Apoptotic process	2.38*E*-13
GO:0042981	Biological process	Regulation of apoptotic process	2.31*E*-07
GO:0097190	Biological process	Apoptotic signaling pathway	2.77*E*-11
GO:0043066	Biological process	Negative regulation of apoptotic process	0.0034509
GO:0019827	Biological process	Stem cell maintenance	0.012077
GO:0032153	Cellular component	Cell division site	0.0080312
GO:0032155	Cellular component	Cell division site part	0.0080312
GO:0000793	Cellular component	Condensed chromosome	1.28*E*-16
GO:0000777	Cellular component	Condensed chromosome kinetochore	2.33*E*-05
GO:0000779	Cellular component	Condensed chromosome, centromeric region	1.56*E*-07
GO:0000794	Cellular component	Condensed nuclear chromosome	1.62*E*-06
GO:0000780	Cellular component	Condensed nuclear chromosome, centromeric region	0.00080836
GO:0000123	Cellular component	Histone acetyltransferase complex	0.00053467
GO:0035097	Cellular component	Histone methyltransferase complex	3.66*E*-06
GO:0044429	Cellular component	Mitochondrial part	1.04*E*-42
GO:0005720	Cellular component	Nuclear heterochromatin	0.00039438
GO:0031519	Cellular component	PcG protein complex	0.008405
GO:0005912	Cellular component	Adherens junction	1.40*E*-13
GO:0003682	Molecular function	Chromatin binding	2.39*E*-08
GO:0031490	Molecular function	Chromatin DNA binding	0.0025282
GO:0042393	Molecular function	Histone binding	8.38*E*-08
GO:0032453	Molecular function	Histone demethylase activity (H3-K4 specific)	0.037622
GO:0004402	Molecular function	Histone acetyltransferase activity	0.019528
GO:0046972	Molecular function	Histone acetyltransferase activity (H4-K16 specific)	0.015756
GO:0043995	Molecular function	Histone acetyltransferase activity (H4-K5 specific)	0.015756
GO:0043996	Molecular function	Histone acetyltransferase activity (H4-K8 specific)	0.015756
GO:0008134	Molecular function	Transcription factor binding	0.0044703

**Notes:**

The details of the table are described below: (1) GO ID: the unique id in gene ontology database; (2) GO category: type of the GO term (cellular component, biological process, or molecular function); (3) description: function description in gene ontology; (4) corrected *P*-value: statistical significance on enrichment with correction.

**Table 2:** Self-renewal and pluripotency related KEGG pathways significantly enriched from DEGs between two mESCs groups.

KEGG ID	KEGG term description	Corrected *P*-value
mmu04110	Cell cycle	6.98*E*-06
mmu04115	p53 signaling pathway	0.008629188
mmu03030	DNA replication	1.73*E*-05
mmu03010	Ribosome	5.67*E*-08
mmu03008	Ribosome biogenesis in eukaryotes	2.00*E*-07
mmu03040	Spliceosome	7.14*E*-11
mmu03015	mRNA surveillance pathway	0.000343
mmu03013	RNA transport	1.66*E*-10
mmu03018	RNA degradation	0.001903
mmu01100	Metabolic pathways	2.00*E*-07
mmu00240	Pyrimidine metabolism	1.25*E*-05
mmu00190	Oxidative phosphorylation	4.13*E*-06
mmu00020	Citrate cycle (TCA cycle)	0.001666
mmu01200	Carbon metabolism	3.66*E*-05

**Notes**:

The details of the table are described below: (1) KEGG ID: unique pathway id in the KEGG database; (2) KEGG term description: function description of the KEGG pathway; (3) Corrected *P*-value: statistical significance of the enrichment with correction.

## Discussion

In current study, the effects of EGFR deficiency on mESCs self-renewal and pluripotency were investigated through its inhibition by AG1478 and bioinformatics analysis of RNA-seq. The results demonstrated the importance of EGFR in maintaining proliferation, self-renewal, and pluripotency of mESCs.

Self-renewal entails fast cell division with a concomitant differentiation suppression, and pluripotency is indispensably associated with this competency of self-renewal. Rapid division of mESCs is associated with a truncated G_1_ phase in cell cycle (Neganova & Lako, 2008). After EGFR inhibition by AG1478, cell cycle of mESCs was significantly arrested at G_0_/G_1_ phase. Expressions of pivotal cell cycle regulatory genes were decreased in EGFR deficient mESCs. The transition from G_1_ phase to S phase were accelerated by CDK2 and CDK4, which formed complex with cyclin E and cyclin D, respectively (Neganova & Lako, 2008), and their expressions were down-regulated after EGFR inhibition. Consistent with the decreased cell viability and S phase length, the expression of PCNA which plays key roles in DNA replication, chromatin remodeling, DNA repair, and cell cycle regulation (Strzalka & Ziemienowicz, 2011) was decreased in EGFR deficient mESCs. GO analysis in EGFR deficient mESCs revealed that many DEGs could be categorized into cell cycle and proliferation related terms, such as cell division site, cell cycle phase transition, and regulation of apoptotic process. Consistently, KEGG enrichment analysis indicated that DEGs were mainly involved in cell cycle and proliferation related pathways, such as cell cycle, DNA replication, and p53 signaling pathway. These gene expression changes may arrested cell cycle at G_0_/G_1_ phase in EGFR deficient mESCs. Thus, EGFR is indispensable in maintaining proliferation and promoting cell cycle progress in mESCs.

Pluripotency factors, such as OCT4, Nanog, and SOX2, are exclusively expressed in undifferentiated mESCs, and play central roles in protecting mESCs self-renewal from differentiation. Once their expressions are decreased, stem cells irresistibly switch from self-renewal to differentiation (Filipczyk et al., 2013; Rizzino, 2013; Young, 2011). The critical roles of pluripotency factors in self-renewal have been demonstrated by the previous results of pluripotency cells researches and induced pluripotent stem cell researches (Morey, Santanach & Di Croce, 2015; Theunissen & Jaenisch, 2014). In our study, expressions of pluripotency factors were dramatically down-regulated in EGFR deficient mESCs, accompanied by decreased expressions of pluripotency markers AP and SSEA-1 as well as increased expressions of early differentiation marker genes, indicating an impairment of self-renewal and occurrence of differentiation in EGFR deficient mESCs. It is well known that LIF plays critical roles in maintaining the self-renewal and pluripotency of mESCs in vitro (Cartwright et al., 2005; Niwa et al., 2009) and Tfcp2l1 was reported as a mediator sensitive to the stimulus of LIF/STAT3 pathway (Ye et al., 2013). We found that Tfcp2l1 expression was dramatically down-regulated in mESCs with EGFR defect, which led to the proposal that there might be lack of mediators to convey extraneous self-renewal stimulus to expression of pluripotency factors. Moreover, the failure of EB formation demonstrated a completely loss of pluripotency in EGFR-inhibited mESCs. Taken together, EGFR deficiency impaired self-renewal and pluripotency in mESCs through decreasing expressions of pluripotency factors.

For better understanding how EGFR inhibition impaired mESCs pluripotency, the transcriptome profiling was performed. Later, the GO analysis showed that significantly enriched GO terms were associated with self-renewal and pluripotency, such as cell cycle, stem cell maintenance, condensed chromosome, chromatin binding, and transcription factor binding. Accordingly, KEGG analysis indicated that DEGs were mainly enriched in pathways of cell cycle, p53 signaling pathway, DNA replication, ribosome, and metabolic regulation. These pathways are related to self-renewal or differentiation initiation. Bioinformatics analysis revealed that EGFR is necessary for self-renewal and pluripotency in mESCs from various aspects, including cell cycle, chromatin structure, epigenetic modification, and metabolic process.

In our study, we found that a switch from self-renewal to differentiation in EGFR-deficient cells. This phenomenon revealed that EGFR is critical for maintaining self-renewal and pluripotency of mESCs. Meanwhile, EGFR inhibition by AG1478 treatment in mESCs markedly reduced cell proliferation, caused cell cycle arrest at G_0_/G_1_ phase, and altered protein expressions of the cell cycle regulatory genes. Zhu et al. reported that cell cycle arrest in G_1_ phase in CNE2 cells induced by AG1478 may be associated with a significant up-regulation of p27 protein levels (Zhu et al., 2001). It has been recently proposed that AG1478 might have EGFR-independent activity in disassembling the Golgi in human cells, through inhibiting the activity of a small GTPase ADP-ribosylation factor (Caja et al., 2011). Therefore, we also need to perform more mechanism studies underlying EGFR function to further elucidate its role in the proliferation, self-renewal, and pluripotency of mESCs.

## Conclusions

Collectively, using the AG1478 inhibition model and RNA-seq analysis, we discovered the importance of EGFR in self-renewal and pluripotency through regulating cell cycle and the expression of pluripotency factors. As a cell surface receptor conveying exogenous stimulations, EGFR deficiency led to cell cycle arrest, and impaired self-renewal and pluripotency. These findings will improve our understanding of the molecular mechanisms driving the self-renewal and pluripotency of mESCs.

## Supplemental Information

10.7717/peerj.6314/supp-1 Supplemental Information 1 Figs. S1–S3 and Tables S1–S6.

10.7717/peerj.6314/supp-2 Supplemental Information 2 Supplemental raw data of Figure 1A and Figure S2A–S2D.

10.7717/peerj.6314/supp-3 Supplemental Information 3 Supplemental raw data of Figure 1D, Figure 2E and 2F, and Figure 4B.

10.7717/peerj.6314/supp-4 Supplemental Information 4 Supplemental raw data of Figure 1B and 1C.

## Additional Information and Declarations

### Data Availability

The following information was supplied regarding data availability: The raw measurements are available in Supplementary Files 1–3.
