# Supplementary material for: EGFR deficiency leads to impaired self-renewal and pluripotency of mouse embryonic stem cells
Source: PeerJ. 2019 Jan 29;7:e6314. doi: 10.7717/peerj.6314 (PMC6357870; doi:10.7717/peerj.6314)
Supplement: Supplemental Information 1 [file peerj-07-6314-s001.docx]

**Supplementary Material**

**
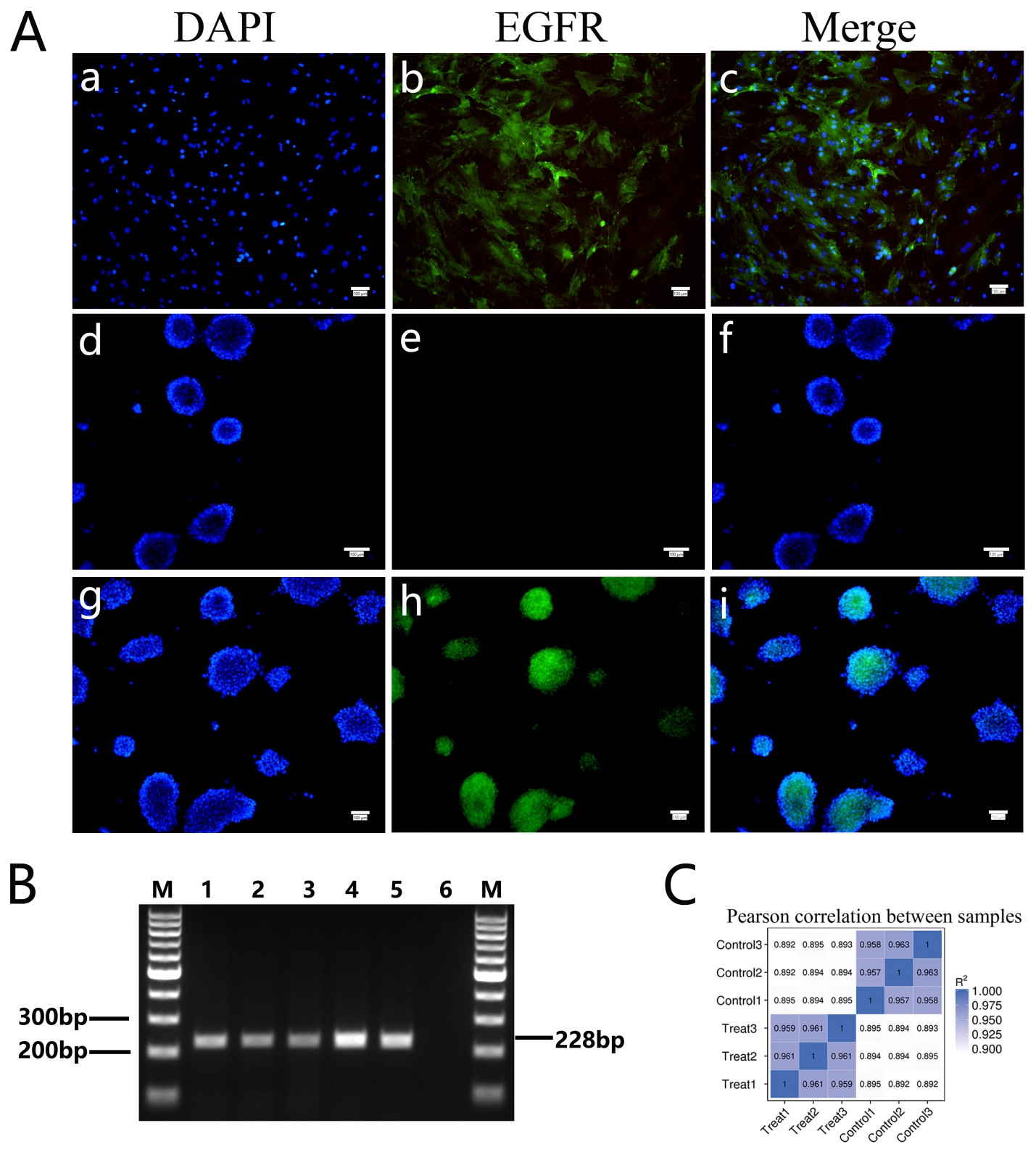
**

**Fig. S1** **Detection of EGFR expression and** **heatmap of correlation coefficients among samples.** (A) The relative distribution of EGFR was investigated by immunofluorescent staining in the mESCs and mouse Leyding cells. a-c. Immunofluorescence images of Leydig cells (positive control) from mouse testis stained with anti-EGFR antibody. d-f. Immunofluorescence images of mESCs (negative control) stained without anti-EGFR antibody. g-i. Immunofluorescence images of mESCs stained with anti-EGFR antibody. Scale bar: 100 µm. (B) The expression of EGFR was investigated by RT-PCR in the mESCs and mouse Leydig cells. Lanes 1-3 represented mESCs. Lanes 4-5 represented mouse Leydig cells. Lane 6 was served as negative control. PCR product length: 228bp. M: 100bp DNA marker. (C) Heatmap of correlation coefficients among samples, R2 means the square of Pearson correlation coefficient.


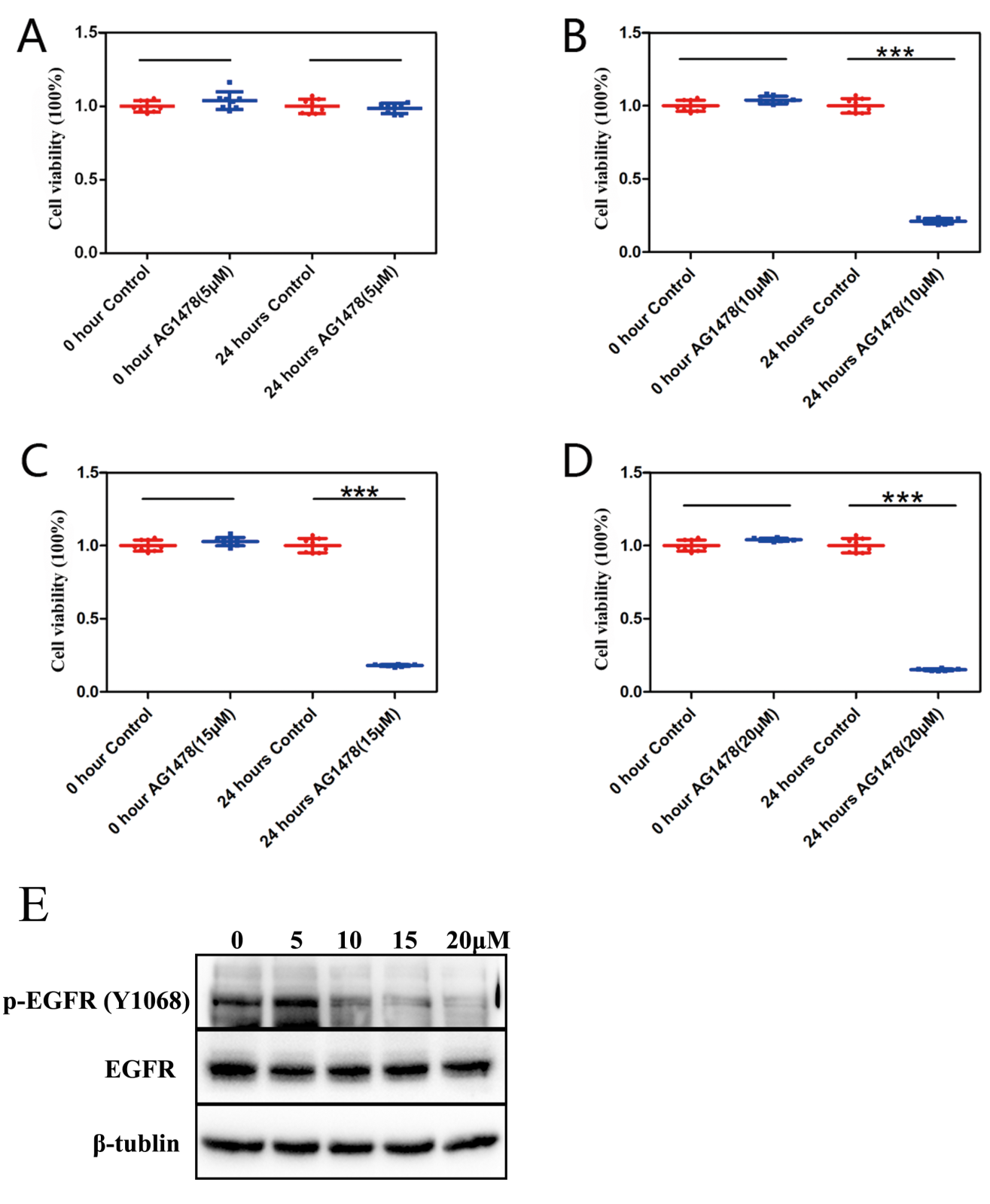


**Fig. S2** **Treatment of mESCs with different concentrations of AG1478 for 24 hours.** Cell viability by CCK-8 assay was performed after mESCs were treated with 5 μΜ (A), 10 μΜ (B), 15 μΜ (C), and 20 μΜ (D) AG1478 for 24 hours. The data are presented as mean ± SD (n = 3; ***P < 0.001, Student’s t-test). (E) Detection of EGFR phosphorylation level in mESCs after AG1478 treatment with different concentrations (5 μΜ, 10 μΜ, 15 μΜ, and 20 μΜ). β-tublin served as a loading control.


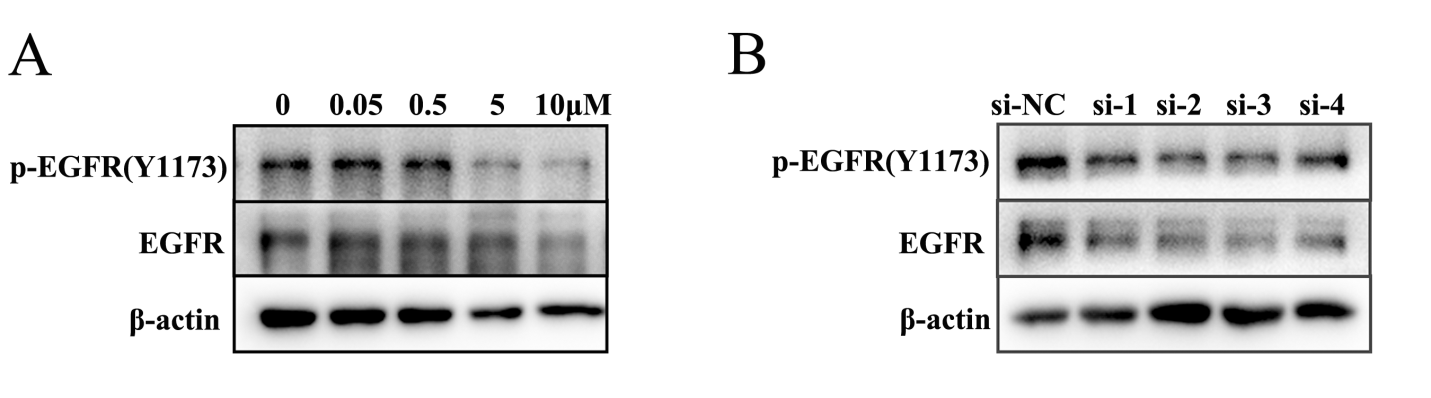


**Fig. S3 Detection of EGFR phosphorylation level in mESCs after** **gefitinib treatment and** **RNA interference.** (A) Detection of EGFR phosphorylation level in mESCs after gefitinib treatment with different concentrations (0.05 μΜ, 0.5 μΜ, 5 μΜ, and 10 μΜ). β-actin served as a loading control. (B) Detection of EGFR phosphorylation level and EGFR expression level in mESCs after RNA interference with different siRNAs (si-Negative Control (NC), si-1, si-2, si-3, and si-4). β-actin served as a loading control.

**Table S1 Primer sequences for cell cycle related genes.**

| **Gene name** | **First primer sequence (5’-3’)** | **Second primer sequence (5’-3’)** |
| --- | --- | --- |
| CyclinA2  CyclinB1  Skp2  Cdc6  Cdc20  PCNA  Cdc25A  CdK1  CdK2  Cdk4  Cul1 | TGCAGCTGTCTCTTTACCCG CAGGCAAGAGTGCCTCTGAA  GCCCTCGGTCCTTTATGGAG  AAAGCGCTGGATGTTTGCAG  AAATGGAGCAGCCTGGAGAC  GAACCTCACCAGCATGTCCA  CATCATGCATGGCAAGCCTC  CCAAGAAGCCGCTTTTCCAC  ATGCAGAGGGGTCCATCAAG  GGGACCTGAAGCCAGAGAAC ACTGAAGTCGAAATTGCTGGT | GTCTGGCTGCCTCTTCATGT GTTTCCATCGGGCTTGGAGA  AAAACCCAGAGCACCCACAA  TCTGCTGGAGAGGAAGGGAA  ACATGGTGTTCTGCTACCCG  AATTCACCCGACGGCATCTT  GAACATCCGCCTTCGCCTTC  TGAGAGCAAATCCAAGCCGT  CATGGTGCTGGGTACACACT CCGACGGAACATCTCTGCAA TCATGATTCTCACGATGGCCG |

**Table S2 Primer sequences for Pluripotency factors.**

| **Gene name** | **First primer sequence (5’-3’)** | **Second primer sequence (5’-3’)** |
| --- | --- | --- |
| Oct4  Nanog  Sox2  C-Myc  Lin28a  Lin28b  Tfcp2l1  PRDM14 | TGTTCAGCCAGACCACCATC GGAACGCCTCATCAATGCCT  ACATGATGGAGACGGAGCTG  TACCTCGTCCGATTCCACGG  CGGAGAAGGCGCCAGAGGAG  GTGCGAGAAGAAGAGTCCAG  TCTTCAAGCAGGAAGAGCCG  CAACCTTGGAAACTGGCAGC | GTGATCTGCTGTAGGGAGGG TGTTCTCCTCCTCCTCAGGG  GCTTGCTGATCTCCGAGTTG  CGACTCCGACCTCTTGGCAG GAACTCCACCGCCTCACCCT CTCCCACTTCTCTTGGTGCC  CGGTCATGGAAAACGACACG ATTTTGGTTGCTGGAACCGC |

**Table S3 Primer sequences for differentiation related genes.**

| **Gene name** | **First primer sequence (5’-3’)** | **Second primer sequence (5’-3’)** |
| --- | --- | --- |
| Notch1 | ACGTAGTCCCACCTGCCTAT | GCCACCTGTGAAGCTGTAG |
| Nestin | GGGGCTACAGGAGTGGAAAC | GACCTCTAGGGTTCCCGTCT |
| Pax6 | TCCAGGTGCTGGACAATGAAA | GGTACAGACCCCCTCGGATA |
| Sox17 | TGCTAGGCAAGTCTTGGAAG | AAAGGCAGACCCAGGCCATC |
| GATA6 | GTGCCTCGACCACTTGCTAT | ATCACTGATGCCCCTACCCC |
| GATA4 | CTGGCGCCTTCATGCACAGC | GGCTCCAGCTTGGCTCCAGC |
| Flk1 | ACTGAGAGATGGGAACCGGA | AGGAGCCAGAAGAACATGGC |
| GSC | GACGAAGTACCCAGACGTGG | CTGTCCGAGTCCAAATCGCT |
| Bmp2 | TGGAAAAGGACATCCGCTCC | AGTTCAGGTGGTCAGCAAGG |

**Table S4 Primer sequences for genes selected to validate RNA-seq data.**

| **Gene name** | **First primer sequence (5’-3’)** | **Second primer sequence (5’-3’)** |
| --- | --- | --- |
| Pramef17  Tdh  Sfn  Cd38  Cdc20  Rab11a  Ddit4  Fabp3  Klf6 | ACTGGCCCTATCCTAGCCTT  AACCTACAACATCAGCGCCA  CTGAACAGGCCGAACGGTAT  GGTCCTGATCGCCTTGGTAG  AAATGGAGCAGCCTGGAGAC ACGTCATCTCAGGGCAGTTC  ACCTGTGTGCCAACCTGATG  CTGGAAGCTAGTGGACAGCA  GTTCGAAGTGGGACCTCTGG | AACTGATCTCCACCCTCTCT  GCATCGTAGCCACCAACTCT  TCGGTCTCTACCTTCTCCCG  AGTGGGGCGTAGTCTTCTCT  ACATGGTGTTCTGCTACCCG TGGCTTGTTCTCAGTGGTGG  CAGCACCAGGGTCAACTGAA  TGGATGAGTTTGCCTCCGTC  CTTGCAAAACGCCACTCACA |

**Table S5 Summary of data production.**

| **Sample name** | **Raw reads** | **Clean reads** | **Clean bases** | **Error rate(%)** | **Q20(%)** | **Q30(%)** | **GC content(%)** |
| --- | --- | --- | --- | --- | --- | --- | --- |
| Control 1 | 101574554 | 99830114 | 14.97G | 0.02 | 95.74 | 89.65 | 49.34 |
| Control 2 | 125533096 | 123372524 | 18.51G | 0.02 | 95.72 | 89.64 | 48.66 |
| Control 3 | 120401168 | 118340812 | 17.75G | 0.02 | 95.73 | 89.66 | 48.77 |
| Treat 1 | 137000172 | 134940612 | 20.24G | 0.02 | 95.85 | 89.82 | 50.08 |
| Treat 2 | 127322850 | 125412030 | 18.81G | 0.02 | 95.81 | 89.68 | 50.1 |
| Treat 3 | 134505802 | 132707222 | 19.91G | 0.02 | 96.07 | 90.13 | 50.64 |

The details of table S5 are described below:
(1) Raw reads: Statistics of raw reads, each adjacent four lines contains the information of one read, and the total read number of each file is calculated;
(2) Clean reads: Same as raw reads, except that only the filtered reads, which all subsequent analysis is based on, is calculated;
(3) Clean bases(G): The product of number and length of sequences, calculated as Giga bases;
(4) Error rate: The error rate of sequencing;
(5) Q20, Q30: The percentage of total number of bases where the Phred score is greater than 20 and 30 which indicates base call accuracy;
(6) GC content: The percentage of G and C in all bases.

**Table S6 Statistics of clean reads mapped to reference genome of mouse.**

| **Sample name** | **Control1** | **Control2** | **Control3** | **Treat1** | **Treat2** | **Treat3** |
| --- | --- | --- | --- | --- | --- | --- |
| Total reads | 99830114 | 123372524 | 118340812 | 134940612 | 125412030 | 132707222 |
| Total mapped | 84356898 (84.5%) | 104688845 (84.86%) | 100589539 (85%) | 109620450 (81.24%) | 102760673 (81.94%) | 109861752 (82.79%) |
| Multiple mapped | 9869132 (9.89%) | 11021012 (8.93%) | 10853702 (9.17%) | 16075988 (11.91%) | 15141683 (12.07%) | 15816371 (11.92%) |
| Uniquely mapped | 74487766 (74.61%) | 93667833 (75.92%) | 89735837 (75.83%) | 93544462 (69.32%) | 87618990 (69.86%) | 94045381 (70.87%) |
| Reads map to '+' | 37270104 (37.33%) | 46854827 (37.98%) | 44899289 (37.94%) | 46810863 (34.69%) | 43856738 (34.97%) | 47084358 (35.48%) |
| Reads map to '-' | 37217662 (37.28%) | 46813006 (37.94%) | 44836548 (37.89%) | 46733599 (34.63%) | 43762252 (34.89%) | 46961023 (35.39%) |
| Non-splice reads | 46884403 (46.96%) | 58689331 (47.57%) | 56546620 (47.78%) | 63097457 (46.76%) | 58922959 (46.98%) | 62907666 (47.4%) |
| Splice reads | 27603363 (27.65%) | 34978502 (28.35%) | 33189217 (28.05%) | 30447005 (22.56%) | 28696031 (22.88%) | 31137715 (23.46%) |

The details of the table S6 are described below:
(1) Total reads: Number of reads after data filtering (clean data);
(2) Total mapped: Number of reads that can be mapped to the genome. Generally, if proper reference genome is available and no contamination during the experimental procedure, the percentage will be higher than 70%;
(3) Multiple mapped: Number of sequences that are mapped to multiple positions in the reference sequences.
(4) Uniquely mapped: Number of reads that are mapped to the unique position in the reference sequences;
(5) Reads map to '+', Reads map to '-': Number of reads that are mapped to the plus or minus strand, respectively.
(6) Splice reads: Number of reads that are mapped to two exons (also known as the junction reads). Similarly, non-splice reads are those that the full-length reads are mapped to one exon. The percentage of splice reads depends on the length of reads.
