# Supplementary material for: EGFR deficiency leads to impaired self-renewal and pluripotency of mouse embryonic stem cells
Source: PeerJ. 2019 Jan 29;7:e6314. doi: 10.7717/peerj.6314 (PMC6357870; doi:10.7717/peerj.6314)

Cell cycle phase distribution of control and AG1478 treated mESCs.

Control 1:


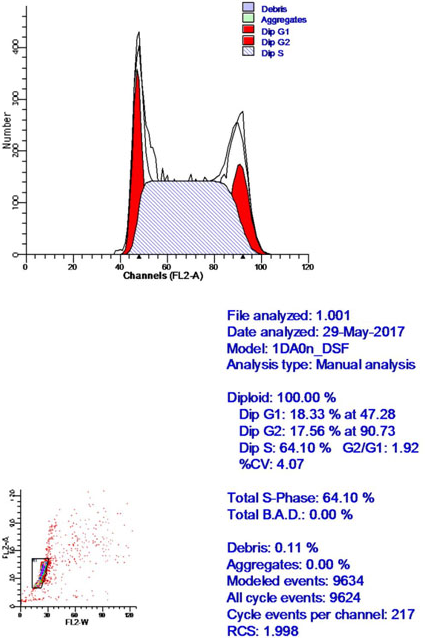


Control 2:


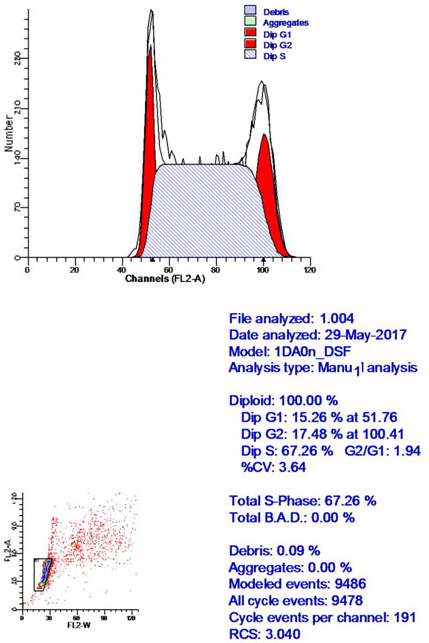


Control 3:


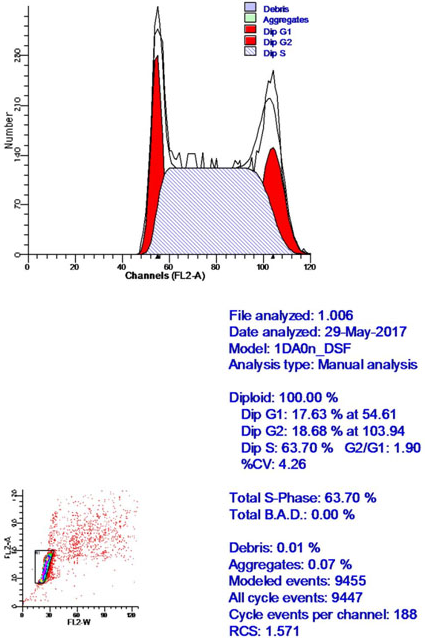


AG1478 treatment 1:


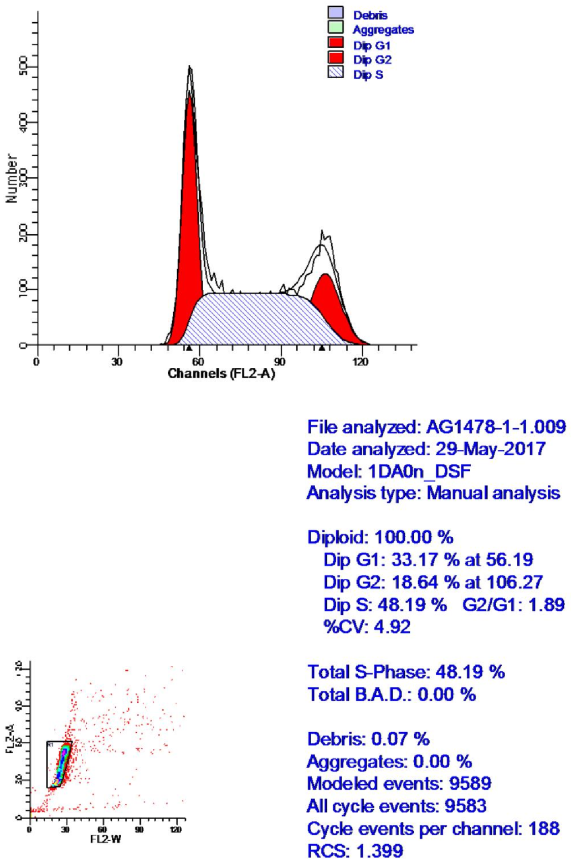


AG1478 treatment 2:


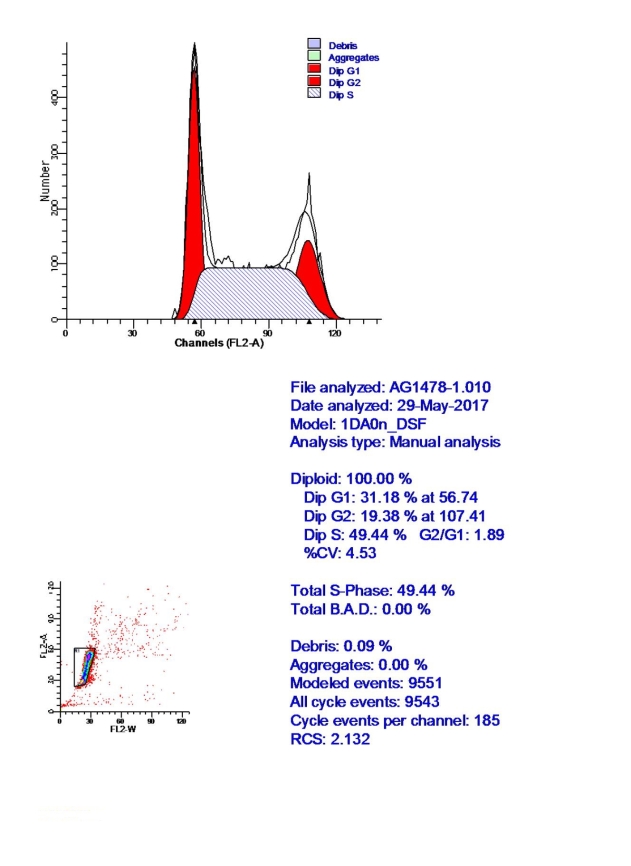


AG1478 treatment 3:


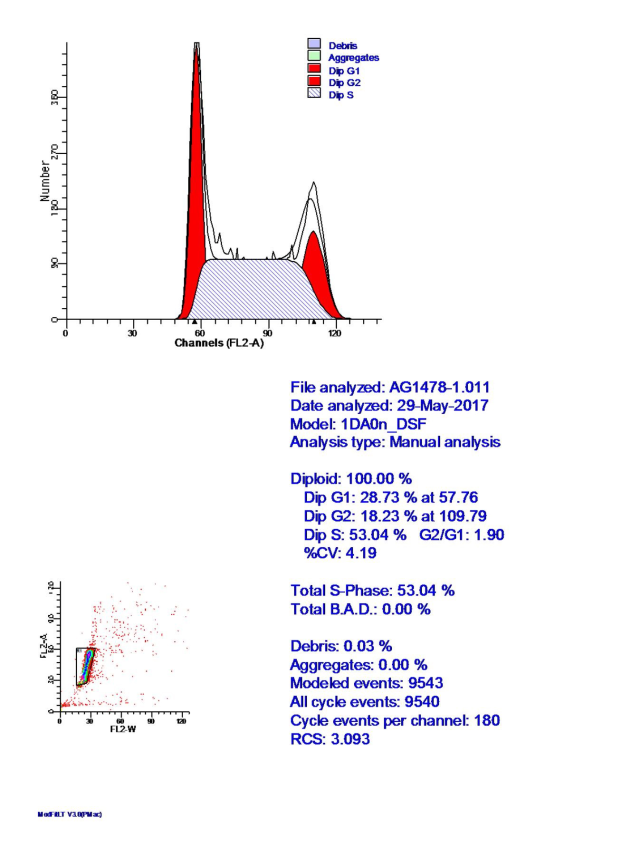

Supplement: Supplemental Information 4 [file peerj-07-6314-s004.docx]
